# Comparative Effects of Including Inorganic, Organic, and Hydroxy Zinc Sources on Growth Development, Egg Quality, Mineral Excretion, and Bone Health of Laying Quails

**DOI:** 10.1007/s12011-024-04137-0

**Published:** 2024-03-04

**Authors:** Osman Olgun, Esra Tuğçe Gül, Gözde Kılınç, Fatih Gökmen, Alpönder Yıldız, Veli Uygur, Ainhoa Sarmiento-García

**Affiliations:** 1 Department of Animal Science, Agriculture Faculty, Selcuk University, 42130 Konya, Turkey; 2 Department of Food Processing, Suluova Vocational Schools, Amasya University, 05500 Amasya, Turkey; 3 Department of Soil Science and Plant Nutrition, Agriculture Faculty, Iğdır University, Iğdır, Turkey; 4 Department of Soil Science and Plant Nutrition, Agriculture Faculty, Applied Sciences University of Isparta, Isparta, Turkey; 5 Área de Producción Animal, Departamento de Construcción y Agronomía, Facultad de Ciencias Agrarias y Ambientales, Universidad de Salamanca, 37007 Salamanca, España

**Keywords:** Zinc Sources, Zinc Hydroxychloride, Bone, Mineral Excretion, Yolk Antioxidant

## Abstract

The purpose of this study was to determine the effects of the dietary addition of zinc (Zn) in the form of sulphate (Zn-S), glycine (Zn-G), and hydroxychloride (Zn-H) on quail performance, eggshell quality, antioxidant status, mineral excretion, biomechanical properties and mineralization of bone. A total of 75 female quails (10-weeks-old) were randomly distributed into 3 groups with 5 replications, each with 5 female quails. Treatment groups were set up by adding Zn-S and Zn-H as the inorganic form and Zn-G as the organic form of zinc to the corn-soybean basal diet (34.14 mg/kg Zn) to obtain 50 mg/kg Zn and the feeding experiment lasted for 12 weeks. Performance parameters and egg production were not impaired by the Zn source (*P* > 0.05). The inclusion of Zn-S in the diet produced a reduction in eggshell thickness, while an improvement in yolk antioxidant capacity (measured as MDA concentration) was detected compared to the other Zn sources (*P* < 0.05). Shear strength and cortical bone area increased (*P* < 0.05) with Zn-G supplementation, however, the mineral concentration of the tibia was higher (*P* < 0.01) in those quails who had received Zn-H. Lower levels of mineral excretion were observed in both types of supplementations compared to Zn-S. Therefore, it can be stated that Zn-G or Zn-H supplementation in the diet of laying quails could be an interesting strategy to reduce mineral excretion and improve bone mineralization without affecting performance compared to Zn-S. However, further studies are needed to determine the differences between Zn-G and Zn-H.

## Introduction

Zinc (Zn) is a crucial trace element to ensure adequate physiological activity of birds [1–3]. Zn is a component of more than 200 metalloenzymes [4], which are involved in several pathways that ensure the proper functioning of the immune system, the development of skeletal tissues, and the maintenance of bone health [5–7]. Zn plays an important role in antioxidant capacities, as it is part of the enzyme superoxide dismutase and other metalloenzymes [5, 8]. In laying and breeding birds, Zn has been associated with improved egg production, since Zn regulates the production of reproductive hormones, and with improved egg quality, since Zn is a component of the enzyme carbonic anhydrase (CA), which is necessary for supplying carbonate ions for eggshell formation [5, 8]. The reason for the improved egg production could be attributed to the impact of zinc on albumin deposition in the formation of eggshells in the uterus (isthmus and magnum region) and the impact of zinc on the potentiation of FSH and LH hormones, progesterone, and oestrogens. The improvement in egg production is also probably due to the impact of zinc on the secretion of reproductive hormones. Estrogen release stimulates oviduct growth and increases protein, vitamins, blood calcium, fats, and nutrients necessary for egg formation [8].

Traditionally, to achieve the nutritional requirements of Zn for birds, plant-based diets are often supplemented with inorganic salts, such as oxides, sulphates, or chlorides, due to relatively low cost [1, 7, 9]. Nevertheless, the inorganic Zn source has limited bioavailability. The ionic bonds of inorganic salts are very weak, enabling the metal ion to fully separate from the sulphate or oxide molecule upon entering into contact with water [10]. This fact causes an interaction with other components of the diet, making their absorption difficult, and resulting in increased trace minerals (TM) excreted into the environment [4]. Hence, the trend in the avian industry has changed toward organic TM, such as proteinate and amino acid forms, based on the higher bioavailability of this form [11]. However, their use has been associated with increased costs in poultry production [9]. To avoid this problem, the use of alternative Zn sources in avian nutrition has become widespread, which enhances availability and absorption as it reduces antagonism in comparison to supplementation with inorganic forms [4, 12]. In this regard, hydroxychloride forms could be an interesting option as a novel inorganic source [9]. This advantage could be explained due to the crystalline structure constituted by stronger covalent bonds between multiple hydroxyl groups and chloride ions rather than carbon-containing ligands as in the ionic forms [10, 13]. Consequently, the solubility of hydroxychloride trace elements is lower in neutral or water solutions and higher in acid solutions, such as the upper part of the small intestine [4, 7]. This results in a lower reactivity of Zn hydroxychloride with other dietary ingredients compared to ionic sources [7, 9, 10]. Hence hydroxychloride forms provide delayed release through digestive processes, improved absorption, and consequently decreased excretion into the environment [12].

Several studies including Zn hydroxychloride in poultry diets have reported improved effects on some traits, including performance and bone health, compared to those receiving inorganic Zn and non-supplemented Zn [9, 10, 12, 14, 15]. In addition, certain studies have proposed that Zn hydroxychloride could enhance some egg attributes as compared to ionic forms [15]. These authors proposed that supplementation with Zn hydroxychloride led to an increase in calcium digestibility and bioavailability, resulting in an elevation of serum calcium level, which presumably was associated with an increase in calcium concentration in the uterine fluid precipitating in the formation of calcite, which facilitated the mineralization of the eggshell. Nevertheless, according to Olouski et al. [12], the data about including hydroxichloride Zn in layer hens’ diet are scarce, and to our knowledge, no available studies are carried out in laying quails. Most of the previous studies [4, 6–10, 12, 14, 15] have conducted comparisons between the addition of dietary two sources of Zn (inorganic vs. organic or traditional vs. novel inorganic sources). To our knowledge, no studies are comparing all three Zn sources.

Japanese quails (*Coturnix coturnix japonic*a) breeding is increasing worldwide, however, the investigations concerning the nutritional aspects of this species are scant [16]. Limited research has been conducted that compares inorganic (traditional or novel) and organic Zn sources in laying diets. Hence, the goal of the current research was to compare the effect of including traditional (Zn-sulphate (Zn-S) novel (Zn-hydroxychloride (Zn-H)) inorganic and organic (Zn-glycine (Zn-G)) Zn sources in laying quail diets on performance, eggshell quality, antioxidant status, bone traits, and mineral excretion.

## Materials and Methods

### Ethical Approval

A special certification was not necessary since the research was carried out on a farm animal. Moreover, the criteria set out in the European Animal Protection Policy [17] as well as the principles described in the 1964 Declaration of Helsinki were complied with throughout the trial period.

### Animal Husbandry

The experiment was performed according to a completely randomized design at a local indoor farm in Selçuklu (Konya, Türkiye) at 38°1′36″, 32°30′45″ coordinates. Seventy-five female Japanese quails (*Coturnix coturnix Japonica*) at the age of 10 weeks, were weighed (253 ± 9.37 g) and randomly assigned to three dietary groups each containing five replicates of five female quails for 12 weeks. All pens had the same measurements (30 × 45 cm) and were well-aired, clean, and sanitized. The room temperature and lighting program were adjusted to 22 °C (± 2.0) and a 16-h respectively. All pens were fitted with separate feeders and drinkers enabling *ad libitum* ingestion of feed and water.

### Dietary Treatments

All quails were fed for 12 weeks with the same basal diet containing corn and soybean meal, with a crude protein content of 200 g/kg crude protein and 2900 kcal/kg metabolizable energy, as can be observed in Table 1. The basal diet submitted in the mash form was developed to fulfil the nutrient requirements of laying quails as provided by the National Research Council [18], including Zn needs which have been established at 50 mg/kg for laying quails.

**Table 1:** Basal diet and its nutrient content (as fed)

Ingredients	g/kg	Nutrient composition	g/kg
Corn	544.0	Metabolizable energy, kcal/kg^2^	2899.08
Soybean meal	344.0	Crude protein^3^	200.13
Soybean oil	36.5	Crude fibre^3^	28.30
Limestone	56.0	Crude fat^3^	58.38
Dicalcium phosphate	11.4	Moisture^3^	128.32
NaCl	3.5	Lysine^2^	10.90
VMP^1^	2.5	Methionine^2^	4.49
DL-methionine	2.1	Cystine^2^	3.73
Total	1000.0	Calcium^3^	24.98
		Total phosphorus^3^	6.37
		Available phosphorus^2^	3.49
		Zinc^3^, mg/kg	34.14

^1^ VMP (vitamin-mineral mixture) as contained per kg: Vitamin-mineral premix (per 1 kg): vitamin A, 8000 IU; vitamin D3, 3000 IU; vitamin E, 5 mg; vitamin K, 2 mg; vitamin B12, 0.02 mg; biotin, 0.1 mg; folic acid, 1 mg; niacin, 50 mg; pantothenic acid, 15 mg; pyridoxine, 4 mg; riboflavin, 10 mg; thiamin, 3 mg; copper, 10 mg; iodine, 1.0 mg; iron, 50 mg; manganese, 60 mg; selenium, 0.42 mg incorporated at 1 g per kg of feed

^2^ Calculated

^3^ Analysed

For that, in all experimental diets, the Zn level was fixed to obtain 50 mg/kg of Zn in the diets, which was supplemented at the expense of corn, to ensure isoproteic and isoenergetic diets. Hence, the experimental groups were as follows: 1) Zn-S (basal diet (34.14 ppm Zn) + 15.86 ppm of Zn-Sulphate), Zn-Glycine (basal diet + 15.86 ppm of Zn-glycine), and 3) Zn-H (basal diet + 15.86 ppm of Zn-hydroxichloride). To determine the chemical composition of the basal diet, the procedures proposed by AOAC [19] were carried out in duplicate. Water content was determined by drying at 105 °C; protein content and fat content were obtained by applying the Kjeldahl method and Soxhlet extraction respectively, while the ash content was obtained by incineration. All those results are listed in Table 1.

### Evaluation of Performance Indicators and Egg Production

To define performance parameters, all experimental quails (*n* = 75) were weighed as a group at the beginning and end of the experiment (± 0.01 g), and average weights of groups were determined. Then, changes in body weight (g) were determined according to the modification of weight. Feed intake (g/quail/day) was estimated as described by Olgun et al. [20] using the total feed provided and the amount left in the feed boxes. To calculate egg production, the number of eggs laid every day was collected, and the value was divided by the total number of birds and multiplied by 100. The outcome was expressed as a percentage (%). Every egg retrieved in the last three days of the study was weighed with a high precision balance (± 0.01 g) to determine egg weight. Egg mass (g/quail/day) and egg-laying efficiency (feed intake/egg mass) were assessed following those described by Sarmiento et al. [16]. The mortality rate also was noted.

### Determination of Eggshell Quality Parameters

A total of 225 eggs, 75 from each treatment, were analysed in the Egg Quality Laboratory (Faculty of Agriculture, Selçuk University, Konya, Türkiye) to evaluate eggshell quality. Eggs collected (*n* = 225) were analysed at the Egg Quality Laboratory (Faculty of Agriculture, Selcuk University, Konya, Türkiye) to evaluate the eggshell quality. At ambient temperature, all eggs gathered in the trial’s final three days were evaluated for internal and external quality standards. Throughout the experiment, broken, damaged, and cracked eggs were listed and expressed as a proportion of the total collected eggs (*n* = 225). The Egg Force Reader (Orka Food Technology Ltd., Ramat Hasharon, Israel) was applied to the blunt part of the egg to measure eggshell strength. Eggshell thickness was determined using a micrometer (Mitutoyo, 0, 01 mm, Japanese) as a medium value (µm) from three separate parts of the shell (equator, blunt, and pointed). The relative eggshell weight (%) has been established by weighing the cleaned and dried shells and dividing them into egg weights.

### Determination of Yolk TBARS and DPPH Values

To evaluate the antioxidant status of the yolk, the malondialdehyde (MDA) and 1-diphenyl-2-picrylhydrazyl (DPPH) concentrations were assessed in triplicate on 75 fresh eggs. To ascertain the MDA value, the thiobarbituric acid reactive substances (TBARS) test was conducted using the modified method described by Kilic and Richards [21] and Sarmiento-García et al. [16]. Measurement of MDA concentration was done quantifying the absorbance value at a wavelength of 530 nm applying a spectrophotometric measurement (Perkin Elmer, USA) based on a blank curve of MDA consisting of 1 mL of trichloroacetic acid (TCA) solution (7.5% TCA, 0.1% EDTA, 0.1% propyl gallate) and 1 mL of thiobarbituric acid (TBA) solution (0.02 M). TBA was expressed as µmol MDA/kg yolk.

The antioxidant ability of the resulting hydrolysates was tested with a modified technique outlined by Sacchetti et al. [22] and Olgun et al. [20] employing the scavenging effect of DPPH radicals. The absorbance measurement was obtained at 517 nm and recorded with a spectrophotometer (Perkin Elmer precisely UV/VIS Spectrometer) by comparison against a blank curve (replaced with 95% ethanol). The Eq. (1) was applied to test the scavenging effect:

$$DPPH\ values = \frac{{[(control\ absorbance - sample\ absorbance)}}{{control\ absorbance}} \times 100$$

### Determination of Bone Biomechanical Properties

After the determination of final body weight, on 84 days, two random female quail from each group (*n* = 30) were randomly selected and euthanized via cervical dislocation. Cortical bone thickness, cortical bone cross-sectional area, shear force, and shear stress of bone were determined following the methods proposed by Gül et al. [23], Armstrong et al. [24], and Wilson and Ruszler [25].

### Faecal and Tibia Mineral Analysis

The mineral content of faecal (*n* = 15) and tibia tissue (*n* = 30) samples was tested according to the wet digestion method, as reported by Olgun and Yıldız [26]. Faeces and tibia samples were dried (105 °C for 24 h), weighed (0.3 ± 0.01 g), and carefully streaked on Teflon-coated digestion plates (Milestone, Sorisole, Bergamo, Italy). Then, each dish was spiked with 5 ml of nitric acid (63.01 M) and 3 ml of perchloric acid (70%). Samples were streaked onto Teflon-coated digestion plates and then introduced into a Mars 5 microwave oven (CEM Corp, 3100 Smith Farm Road, Matthews, NC, USA) at 190 °C for 40 min. Following the completion of the incineration process, the supernatant solution contained inside the Teflon-coated sample containers was subjected to a dilution of 50 ml using distilled water. Thereafter, 0.1 g of each ash sample was weighed to establish the mineral content, comprising the concentration of Cu, calcium, phosphorus, manganese, and zinc, using inductively coupled plasma atomic emission spectrometry (ICP-OES) (Thermo Scientific 7200 ICP-OES Analyser, Thermofisher Scientific, Waltham, USA).

### Statistical Analysis

All data were assessed using one-way ANOVA, which was performed using the SPSS 22.0 software package (SPSS Inc., Chicago, IL, USA) considering the cage as the test unit. Each cage means was used as the experimental unit for growth performance measurements. For other measurements (i.e., egg quality, MDA, DPPH, etc.), the individual bird was used as the experimental unit. The results obtained were shown in the following Tables, as means ± standard errors of the mean (SEM). Statistical significance was assumed as a probability value of *p* < 0.05, while a probability value of *p* < 0.10 was defined as a trend.

## Results

### Performance Parameters

Table 2 demonstrates the effect of using different forms of Zn in the diet on the performance of quails. The Zn supplementation source was not observed to have any effect (*P* > 0.05) on the performance parameters, including final body weight, body weight change, feed intake, and egg-laying efficiency. Similar results were described for egg production parameters which remained unaffected regardless of the Zn source (*P* > 0.05).

**Table 2:** Effect of different zinc sources on performance (*n* = 75) in laying quails

Parameters	Zn-S	Zn-G	Zn-H	S.E.M	*P*-values
Initial body weight (g)	251.38	254.75	252.25	2.263	0.841
Final body weight (g)	264.38	276.00	265.63	3.378	0.327
Body weight change (g)	13.00	21.25	13.38	3.758	0.634
Egg production (per egg/100 quails)	91.87	92.86	91.19	0.556	0.503
Egg weight (g)	12.93	13.37	13.20	0.094	0.151
Egg mass (g/quail/day)	11.87	12.42	12.04	0.119	0.166
Feed intake (g/quail/day)	32.85	33.17	31.40	0.356	0.086
Egg-laying efficiency	2.77	2.67	2.61	0.036	0.198

S.E.M.: Standard error means. Zn-S: Zinc sulphate, Zn-G: Zinc glycine, Zn-H: Zinc hydroxychloride

### Eggshell Quality

In the study, the damaged egg rate, eggshell breaking strength, eggshell weight, and thickness were examined as quality parameters and are demonstrated in Table 3. There was no effect of Zn source on the broken egg rate, eggshell breaking strength, and eggshell weight (*P* > 0.05). These values range from 0.00 to 1.52% for damaged egg rate, from 12.02 to 12.78 N for eggshell breaking strength, and from 8.37 to 8.61% for eggshell weight. Nevertheless, the dietary Zn source in the diet affected the eggshell thickness statistically (*P* < 0.05), and the value increased by dietary using Zn-G (225.46 μm) or Zn-H (224.55 μm) instead of Zn-S (217.08 μm).

**Table 3:** Eggshell quality (*n* = 225) of laying quails fed with three different Zn sources

Parameters	Zn-S	Zn-G	Zn-H	S.E.M	*P*-values
Damaged egg (per egg/100 eggs)	0.32	0.00	1.52	0.404	0.292
Eggshell-breaking strength (N)	12.02	12.38	12.78	0.167	0.182
Relative eggshell weight (g shell/ 100 g egg)	8.37	8.37	8.61	0.066	0.236
Eggshell thickness (µm)	217.08^b^	225.46^a^	224.55^a^	1.381	0.011

S.E.M.: Standard error means, Zn-S: Zinc sulphate, Zn-G: Zinc glycine, Zn-H: Zinc hydroxychloride, ^a,b^: Means with different upper letters in the same row are different at the *P* < 0.05 level

### Yolk Antioxidant Capacity

The effect of including different dietary Zn sources on the antioxidant capacity of the yolk measured as DPPH and MDA values was given in Table 4. Yolk DPPH value was reduced (*P* < 0.001) in the group that had received Zn-H (6.235%) than those fed with Zn-S and Zn-G (8.126 and 8.802% respectively). Contrarily, the MDA value was found to be significantly decreased (*P* < 0.01) in the quails feed with Zn-S (1.537 µmol/kg) than in those receiving Zn-G (2.394 µmol/kg) or Zn-H (2.285 µmol/kg).

**Table 4:** Effect of different zinc sources on DPPH and and MDA values of yolk (*n* = 75) in laying quails

Parameters	Zn-S	Zn-G	Zn-H	S.E.M	*P*-values
DPPH (% reducing)	8.126^a^	8.802^a^	6.235^b^	0.2532	< 0.001
MDA value (µmol MDA/kg)	1.537^b^	2.394^a^	2.285^a^	0.1297	0.009

DPPH: 2,2-diphenyl-1-picrylhydrazyl, MDA: Malondialdehyde, S.E.M.: Standard error means, Zn-S: Zinc sulphate, Zn-G: Zinc glycine, Zn-H: Zinc hydroxychloride, ^a,b^: Means with different upper letters in the same row are different at the *P* < 0.05 level

### Mineral Excretion

Table 5 shows the effect of using different Zn dietary sources on mineral excretion in faeces. In general, all minerals evaluated in the faeces were affected by the dietary sources of Zn, except for phosphorus content, which showed similar values for all experimental diets, ranging from 1.82 to 1.93% (*P* > 0.05).

**Table 5:** Mineral faecal excretion (*n* = 15) of laying quails fed with three different Zn sources

Parameters	Zn-S	Zn-G	Zn-H	S.E.M	*P*-values
Zinc (mg/kg)	233.68^a^	190.23^b^	151.77^c^	9.756	< 0.001
Copper (mg/kg)	37.72^a^	23.18^c^	27.68^b^	1.705	< 0.001
Manganese (mg/kg)	390.20^a^	283.94^c^	331.79^b^	13.958	0.001
Calcium (%)	4.35^b^	4.46^b^	5.52^a^	0.209	0.027
Phosphorus (%)	1.82	1.93	1.91	0.049	0.658

S.E.M.: Standard error means, Zn-S: Zinc sulphate, Zn-G: Zinc glycine, Zn-H: Zinc hydroxychloride, ^a,b,c^: Means with different upper letters in the same row are different at the *P* < 0.05 level

The Zn excretion was highest (*P* < 0.01) in the group using Zn-S (233.68 mg/kg), followed by Zn-G (190.23 mg/kg) and Zn-H (151.77 mg/kg). Similar behaviour was described for faecal excretions of copper (*P* < 0.01) and manganese (*P* < 0.01). Was reported an increased value in the quails fed with Zn-S (37.72 and 390.20 mg/kg, respectively) compared to those received Zn-H (27.68 and 331.79 mg/kg, respectively) while the group that had been supplemented with Zn-G reported intermediate value (23.18 and 283.94 mg/kg, respectively). Finally, the quails supplemented with Zn-H excreted higher amounts of calcium (5.52%) than those fed with Zn-S (4.35%) or Zn-G (4.46%).

### Bone Biomechanical Traits

The potential effects of different dietary Zn sources on bone biomechanical parameters of laying quails are shown in Table 6. There was no effect (*P* > 0.05) of Zn dietary source on cortical bone thickness (0.320–0.358 mm) and shear stress (101.32–108.60 N/mm^2^). Nevertheless, regarding the cortical bone cross-sectional area and shear force of bone differences were reported depending on the dietary Zn source. In both cases, significantly (*P* < 0.05) higher values were found in the quails supplemented with Zn-G (1.39 mm^2^ and 140.40 N, respectively) than those received Zn-S (1.17 mm^2^ and 125.48 N, respectively) or Zn-H (1.20 mm^2^ and 127.61 N, respectively).

**Table 6:** Effect of different zinc sources on biomechanical traits of tibia (*n* = 30) in laying quails

Parameters	Zn-S	Zn-G	Zn-H	S.E.M	*P*-values
Cortical bone thickness (mm)	0.329	0.358	0.320	0.0077	0.112
Cortical bone cross-sectional area (mm^2^)	1.17^b^	1.39^a^	1.20^b^	0.041	0.043
Shear force (N)	125.48^b^	140.40^a^	127.61^b^	2.509	0.017
Shear stress (N/mm^2^)	107.86	101.32	108.60	2.619	0.497

S.E.M.: Standard error means, Zn-S: Zinc sulphate, Zn-G: Zinc glycine, Zn-H: Zinc hydroxychloride, ^a,b^: Means with different upper letters in the same row are different at the *P* < 0.05 level

### Bone Mineralization

The effect of the addition of Zn dietary source on the mineral bone content of laying quails was given in Table 7. Zn dietary sources did not alter (*P* > 0.05) the calcium (24.69–27.10%) and magnesium (12.74–15.75 mg/kg) concentrations in the bone. Nevertheless, adding Zn-H to the laying quails’ diet resulted in an improved (*P* < 0.01) concentration of Zn, Cooper, and Phosphorus as compared to those fed with Zn-G or Zn-S.

**Table 7:** Effect of different zinc sources on mineral contents of tibia (*n* = 30) in laying quails

Parameters	Zn-S	Zn-G	Zn-H	S.E.M	*P*-values
Zinc (mg/kg)	310.02^b^	314.48^b^	342.39^a^	4.798	0.002
Copper (mg/kg)	1.62^b^	1.33^b^	2.78^a^	0.179	< 0.001
Manganese (mg/kg)	12.74	15.75	14.54	0.903	0.421
Calcium (%)	26.62	27.10	24.69	0.668	0.318
Phosphorus (%)	14.14^b^	14.81^b^	17.14^a^	0.429	0.002

S.E.M.: Standard error means, Zn-S: Zinc sulphate, Zn-G: Zinc glycine, Zn-H: Zinc hydroxychloride, ^a,b^: Means with different upper letters in the same row are different at the *P* < 0.05 level

## Discussion

This research indicated that none of the assessed dietary Zn sources impaired the performance of laying quails. Similar results were described for egg production and egg weight, which were not affected by the source of Zn in the diet. These results were consistent with those described by previous authors [1, 11, 27, 28] when hens were fed with different dietary Zn sources. The results of the current research suggest that if dietary Zn requirements are met without stress conditions, the Zn dietary source would not impair quail development and egg production, as reported by Franklin et al. [29]. Contrary, Olukosi et al. [12] described a marginal increase in egg-laying efficiency for the hens fed with hydroxichloride trace minerals (TM) compared to those received sulphate TM, although these authors justify it due to the enhanced feed intake. Similarly, van Kuijk et al. [30] described an improvement effect on final body weight and body weight gain of broilers fed with hydroxichloride TM than those supplemented with sulphate. Jiang et al. [15] showed a higher value for the egg-laying efficiency and egg weight in the hens fed with hydroxichloride TM compared to the sulphate form. The lack of differences in the current research may be because previous studies evaluated the effect of the combination of TM (Zn, copper, and manganese), and not a single TM (Zn) as in this study. TM plays a key role in avian nutrition to guarantee efficient production and development [12]. The mixture of TM could improve protein synthesis and deposition in the albumen, as well as cell multiplication and division, which could improve egg weight and egg production. In addition, all these minerals are involved in different metabolic processes (including those related to obtained DNA, carbohydrates, and proteins) that would explain the improvement in performance parameters [15].

Eggshell quality is still an important issue for poultry farming, as it entails great economic losses for poultry producers [23]. Even though no differences in egg damage, eggshell-breaking strength, or relative eggshell weight were described depending on the experimental sources of Zn provided to the quails, a positive effect on eggshell thickness was described in quails fed with Zn-G or Zn-H compared to those received Zn-S. According to previous authors, the substitution of inorganic with hydroxychloride [12, 15] or organic [28, 31, 32] Zn sources in the hen diet could result in the enhancement of some eggshell traits. It has been described that hydroxichloride sources lead to an increase in the calcium content of blood, which presumably raises the level of calcium in the uterine fluid and uterine glands resulting in efficient eggshell mineralization which affects the eggshell thickness [31]. Similarly, organic Zn sources have been linked with an enhancement in the capacity of the shell gland to produce Zn [28]. Since Zn is an essential constituent of carbonic anhydrase (CA) found in the cell membranes of tubular glands and capillary endothelium, and its deficiency is associated with the development of thinner-shelled eggs, reduced absorption of Zn is likely to result in decreased eggshell thickness [15]. Those results, raise the hypothesis of greater digestibility and availability of Zinc when quails had fed with hydroxichloride (Zn-H) and organic sources (Zn-G), compared to those receiving the inorganic sources (Zn-S) leading to an enhanced eggshell thickness.

Zn is a cofactor of Cu Zn-superoxide dismutase, which plays a key role in suppressing free radicals and inhibiting NADPH-dependent lipid peroxidation, which reduces lipid peroxidation and enhances the antioxidant capacity [15]. Contrary to expectations, supplementation with Zn-H in the diet increased the MDA value and decreased the DPPH value of the yolk. It was expected that, due to a higher bioavailability of Zn in quails supplemented with Zn-G or Zn-H, this would result in a higher deposition in the yolk, with a consequent improvement of the antioxidant capacity of the egg. Even though no research has been conducted to compare the effect of Zn sources on the antioxidant or oxidative stress characteristics of egg yolks, previous research has stated the effect of Zn sources on certain antioxidant markers. For example, previous studies comparing dietary supplementation with Zn-S and Zn-G as inorganic and organic forms, respectively, did not observe changes in antioxidant capacity measured as plasma, hepatic and renal MDA [33], hepatic MDA [27] or serum SOD. According to Jiang et al. [15], laying hens’ serum SOD was unaffected by the sulphate and hydroxychloride forms of zinc, copper, and manganese. Similarly, Yu et al. [9] found that Zn forms (Zn-S and Zn-H) did not affect serum T-SOD, ZnCu-SOD, and MDA values of broilers. In contrast to the current study, Olukosi et al. [12] stated that the serum MDA in broilers was better in the Zn-H group than in Zn-S. The difference between the studies could probably be due to the different methods used to evaluate the antioxidant capacity, as well as the tissue used. Furthermore, the age of the bird, the species used, and the duration of the assay may be responsible for the differences found [31]. Therefore, further studies are needed to evaluate the mechanisms of the antioxidant capacity of the yolk that has been produced.

The poultry industry has focused on the search for nutritional strategies that meet the TM needs of poultry without increasing TM residues in faeces, which are harmful to the environment [11]. Interesting results were observed in the faecal excretion of minerals as a function of dietary Zn source. Regarding Zn excretion, the lowest faecal excretion of Zn was recorded in the group supplemented with Zn-H, followed by the group that had received Zn-G, and, finally, the highest value of excretal Zn was recorded in the group fed with Zn-S. While the lowest Cu and Mn excretion was recorded for the quails supplemented with Zn-G. To our knowledge, the previous studies did not compare all the Zn sources assessed in this research. Nevertheless, it seems clear that traditional inorganic TM sources result in a higher mineral excretion than other dietary TM sources. Han et al. [28] explained that organic minerals improved Zn absorption which resulted in a lower Zn excretion as compared to inorganic sources such as sulphate, which is consistent with the results of the current research. The higher bioavailability of Zn-G chelates compared to Zn-S could be linked to the chelates’ ring structure, which preserves Zn against chemical reactions in the digestive tract and maintains its stability despite the low pH. Moreover, chelates are negatively loaded to promote efficient passage through the intestinal wall by several pathways (mainly by amino acid transport) in comparison to inorganic zinc. Similar findings have been reported by Villagómez-Estrada et al. [7] who described a lower faecal excretion (Zn, Mn, Cu) when pigs were supplemented with hydroxychloride instead of inorganic TM. In this sense, inorganic TM tends to break down and interact with other dietary components in the upper gastrointestinal tract, compromising their bioavailability and leading to increased excretion [12]. However, the differences found in this study between the Zn-G and Zn-H forms have not been previously documented in quail. Hence, the results obtained in the current research could be better explained by further investigating the bioavailability of Zn-H concerning organic forms.

Bone shear force and collagen crosslink content are strongly correlated, and Zn is essential for collagen synthesis. Furthermore, Zn supplementation has been found to enhance the anabolic action of insulin-like growth factor I on osteoblasts, thus having a positive influence on bone formation [10]. All the parameters evaluated on the biomechanical characteristics of the tibia were identical in quails supplemented with Zn-S and those supplemented with Zn-H. These results are partially supported by the reports of Nguyen et al. [10], who stated that there is no difference between Zn hydroxychloride and a combination of oxides and sulphates concerning the effect on bone strength of broilers when the same doses are used. However, in the current research quails supplemented with Zn-G showed higher values for cortical bone cross-sectional area and shear strength. Those findings could suggest an improved collagen crosslink content and osteoblast growth leading to an enhanced bone status [10]. However, there seems to be no agreement among authors on the effect of different forms of Zn on bone biomechanical parameters. Olgun and Yıldız [1] expressed that using Zn-S in laying hens was effective in improving bone strength compared to the use of Zn-G. While Cufadar et al. [11] and Niknia et al. [8] stated that organic or inorganic Zn forms did not affect bone biomechanical properties in hens. Niknia et al. [8] stated that high doses are necessary to improve bone biomechanical parameters (up to 100 mg/kg in broilers). They also speculated that the improvements are less evident as the age of the animals increases. In any case, further studies are needed to verify the improvement of these parameters associated with Zn-G supplementation.

In the current study, the tibia Zn, copper, and phosphorus contents of the quails supplemented with Zn-H were found to be higher than the groups using Zn-S or Zn-G. According to the results of this study, it is observed that there is a negative correlation between Zn accumulation and Zn excretion in the tibia. Therefore, high Zn retention with the Zn-H form may have increased the accumulation of this mineral in bone which is in line with those described by Pérez et al. [34]. These authors stated that hydroxichloride forms tend to be less reactive with other minerals and vitamins contained in the feed. Similar findings were reported by Ülger and Mahmood [35], who described an enhancement in Zn serum when nano-Zn (6 mg/kg) was added to the laying quails’ diet. This would be responsible for improved absorption of Zn and other micronutrients, like vitamin A, enhancing the absorption of trace minerals. Contrary to the current research, previous authors have stated that the Zn source did not affect the mineral content of the tibia. For example, Nguyen et al. [10] found that there was no difference between supplementation with Zn hydroxychloride, oxide, and sulfate on bone mineralization (including Zn, calcium, and phosphorus). Olukosi et al. [4] reported that the use of sulphate or hydroxychloride forms of Zn in the diet did not affect the Zn and Cu content of broilers´ bones. Similarly, Villagómez-Estrada et al. [7] reported that feeding pigs with Zn-S and Zn-H did not modify Zn and copper concentrations in pig serum, liver and bone, which are in line with those stated by Niknia et al. [8] in aged hens. Olgun and Yıldız [1] demonstrated that the use of Zn-S and Zn-G did not cause any difference in tibia Zn, calcium, and phosphorus levels. The Zn supplementation levels, physiological state, age, and type of birds may be the reason for the differences between previous studies. In any case, further research is needed to re-evaluate these results.

## Conclusions

In summary, the results of the current research demonstrate that none of the assessed Zn sources compromise the performance or egg production of laying quails. Regarding eggshell quality, Zn-S showed a decrease in eggshell thickness, while an enhancement in the antioxidant capacity (measured as DPPH and MDA value) was recorded. It is important to highlight those quails supplemented with Zn-H reduced the amounts of Zn, Cu, and Mn excreted in the faeces to the environment as the increased mineral content of the bones compared with those supplemented with Zn-S. Based on these results, it can be concluded that Zn-H supplementation in the diet of laying quails is a suitable option to reduce TM excretion to the environment, leading to an increase in bone TM without affecting performance in comparison with Zn-S. However, the comparison between the improvements derived from Zn-G and Zn-H supplementation does not seem clear, and further studies are needed.

## Data Availability

No datasets were generated or analysed during the current study.

## References

[1] Olgun O, Yildiz AÖ (2017) Effects of dietary supplementation of inorganic, organic or nano zinc forms on performance, eggshell quality, and bone characteristics in laying hens. Ann Anim Sci 17:463–476. 10.1515/aoas-2016-0055

[2] Broom LJ, Monteiro A, Piñon A (2021) Recent advances in understanding the influence of zinc, copper, and manganese on the gastrointestinal environment of pigs and poultry. Animals 11:1276. 10.3390/ani11051276 33946674 10.3390/ani11051276 PMC8145729

[3] Sahin N, Kucuk O, Orhan C, Savasli E, Cakmak I, Sahin K (2022) Feeding zinc-biofortified wheat improves performance, nutrient digestibility, and concentrations of blood and tissue minerals in quails. Biol Trace Elem Res 200:3774–3784. 10.1007/s12011-021-02955-0 34637103 10.1007/s12011-021-02955-0 PMC8505784

[4] Olukosi OA, Kujik SV, Han Y (2018) Copper and zinc sources and levels of zinc inclusion influence growth performance, tissue trace mineral content, and carcass yield of broiler chickens. Poult Sci 97:3891–3898. 10.3382/ps/pey247 29982614 10.3382/ps/pey247 PMC6162356

[5] Huang L, Li X, Wang W, Yang L, Zhu Y (2019) The role of zinc in poultry breeder and hen nutrition: an update. Biol Trace Elem Res 192:308–318. 10.1007/s12011-019-1659-0 30767181 10.1007/s12011-019-1659-0

[6] Yu Q, Liu H, Yang K, Tang X, Chen S, Ajuwon KM, Degen A, Fang R (2020) Effect of the level and source of supplementary dietary zinc on egg production, quality, and zinc content and on serum antioxidant parameters and zinc concentration in laying hens. Poult Sci 99:6233–6238. 10.1016/j.psj.2020.06.029 33142541 10.1016/j.psj.2020.06.029 PMC7647701

[7] Villagómez-Estrada S, Pérez JF, van Kuijk S, Melo-Durán D, Karimirad R, Solà-Oriol D (2021) Effects of two zinc supplementation levels and two zinc and copper sources with different solubility characteristics on the growth performance, carcass characteristics and digestibility of growing-finishing pigs. J Anim Physiol Anim Nutr 105:59–71. 10.1111/jpn.13447 10.1111/jpn.13447 PMC7821212 32969109

[8] Niknia AD, Vakili R, Tahmasbi AM (2022) Zinc supplementation improves antioxidant status, and organic zinc is more efficient than inorganic zinc in improving the bone strength of aged laying hens. Vet Med Sci 8:2040–2049. 10.1002/vms3.896 35925611 10.1002/vms3.896 PMC9514485

[9] Yu L, Yi J, Chen Y, Huang M, Zhu N (2022) Relative bioavailability of broiler chickens fed with zinc hydroxychloride and sulphate sources for corn-soybean meal. Biol Trace Elem Res 200:4114–4125. 10.1007/s12011-021-03013-5 34825318 10.1007/s12011-021-03013-5

[10] Nguyen HTT, Morgan N, Roberts JR, Wu SB, Swick RA, Toghyani M (2021) Zinc hydroxychloride supplementation improves tibia bone development and intestinal health of broiler chickens. Poult Sci 100:101254. 10.1016/j.psj.2021.101254 34174567 10.1016/j.psj.2021.101254 PMC8242038

[11] Cufadar Y, Göçmen R, Kanbur G, Yıldırım B (2020) Effects of dietary different levels of nano, organic and inorganic zinc sources on performance, eggshell quality, bone mechanical parameters and mineral contents of the tibia, liver, serum and excreta in laying hens. Biol Trace Elem Res 193:241–251. 10.1007/s12011-019-01698-3 30941677 10.1007/s12011-019-01698-3

[12] Olukosi OA, Van Kuijk SJ, Han Y (2019) Sulphate and hydroxychloride trace minerals in poultry diets–comparative effects on egg production and quality in laying hens, and growth performance and oxidative stress response in broilers. Poult Sci 98:4961–4971. 10.3382/ps/pez261 31075168 10.3382/ps/pez261 PMC6748738

[13] Cemin HS, Woodworth JC, Tokach MD, Dritz SS, DeRouchey JM, Goodband RD (2017) Effect of zinc oxide, zinc hydroxychloride, and tri-basic copper chloride on nursery pig performance. Kans Agric Exp Stn Res Rep 3(7):1–9. 10.4148/2378-5977.7482

[14] Dos Santos TS, Augusto KVZ, Han Y, Sartori MMP, Denadai JC, Santos CT, Sobral NC, Roça RO, Sartori JR (2021) High levels of copper and zinc supplementation in broiler diets on growth performance, carcase traits, and apparent ileal mineral absorption. Brit Poult Sci 62:579–588. 10.1080/00071668.2021.1887453 33555207 10.1080/00071668.2021.1887453

[15] Jiang Q, Sun J, He Y, Ma Y, Zhang B, Han Y, Wu Y (2021) Hydroxychloride trace elements improved eggshell quality partly by modulating uterus histological structure and inflammatory cytokines expression in aged laying hens. Poult Sci 100:101453. 10.1016/j.psj.2021.101453 34624774 10.1016/j.psj.2021.101453 PMC8503664

[16] Sarmiento-Garcia A, Olgun O, Kılınç G, Sevim B, Gökmen SA (2023) The use of purple carrot powder in the diet of laying quails improved some egg quality characteristics, including antioxidant capacity. Trop Anim Health Produc 55:220. 10.1007/s11250-023-03636-x 10.1007/s11250-023-03636-x PMC10205830 37221423

[17] EPCEU-The European Parliament and the Council of the European Union (2010) Directive 2010/63/EU of the European Parliament and of the Council of 22 September 2010 on the Protection of Animals Used for Scientific Purposes; European Parliament: Strasbourg, France, pp. 1–47

[18] National Research Council (1994) Nutrient requirements of Poultry. Nutrient requirements of Poultry. National Academies

[19] AOAC (2005) Animal Feed. In: Official Methods of Analysis. 18th Edition, Association of Official Analytical Chemists, Gaithersburg, USA, pp 27–48

[20] Olgun O, Gül ET, Kılınç G, Yıldız A, Çolak A, Sarmiento-García A (2022) Performance, egg quality, and yolk antioxidant capacity of the laying quail in response to dietary choline levels. Animals 12:3361. 10.3390/ani12 36496882 10.3390/ani12233361 PMC9735531

[21] Kilic B, Richards MP (2003) Lipid oxidation in poultry döner kebab: pro-oxidative and anti-oxidative factors. J Food Sci 68:686–689. 10.1111/j.1365-2621.2003.tb05732.x

[22] Sacchetti G, Maietti S, Muzzoli M, Scaglianti M, Manfredini S, Radice M, Bruni R (2005) Comparative evaluation of 11 essential oils of different origin as functional antioxidants, antiradicals and antimicrobials in foods. Food Chem 91:621–632. 10.1016/j.foodc

[23] Gül ET, Olgun O, Yıldız A, Tüzün AE, Sarmiento-García A (2022) Use of maca powder (*Lepidium meyenii*) as feed additive in diets of laying quails at different ages: its effect on performance, eggshell quality, serum, ileum, and bone properties. Vet Sci 9:418. 10.3390/vetsci9080418 36006333 10.3390/vetsci9080418 PMC9415308

[24] Armstrong TA, Flowers WL, Spears JW, Nielsent FH (2002) Long-term effects of boron supplementation on reproductive characteristics and bone mechanical properties in gilts. J Anim Sci 80:154–161. 10.2527/2002.801154x 11831513 10.2527/2002.801154x

[25] Wilson JH, Ruszler PL (1996) Effects of dietary boron supplementation on laying hens. Brit Poult Sci 37:723–729. 10.1080/00071669608417902 8894217 10.1080/00071669608417902

[26] Olgun O, Yıldız AÖ (2014) Effect of dietary supplementation of essential oils mixture on performance, eggshell quality, hatchability, and mineral excretion in quail breeders. Environ Sci Pollut Res 21:13434–13439. 10.1007/s11356-014-3285-x 10.1007/s11356-014-3285-x 25012208

[27] Zhang L, Wang YX, Xiao X, Wang JS, Wang Q, Li KX, Guo TY, Zhan XA (2017) Effects of zinc glycinate on productive and reproductive performance, zinc concentration and antioxidant status in broiler breeders. Biol Trace Elem Res 178:320–326. 10.1007/s12011-016-0928-4 28130743 10.1007/s12011-016-0928-4

[28] Han Q, Guo Y, Zhang B, Nie W (2020) Effects of dietary zinc on performance, zinc transporters expression, and immune response of aged laying hens. Biol Trace Elem Res 196:231–242. 10.1007/s12011-019-01916-y 31773485 10.1007/s12011-019-01916-y

[29] Franklin SB, Young MB, Ciacciariello M (2022) The impact of different sources of zinc, manganese, and copper on broiler performance and excreta output. Animals 12:1067. 10.3390/ani12091067 35565494 10.3390/ani12091067 PMC9103251

[30] Van Kuijk SJA, Han Y, Garcia-Ruiz AI, Rodiles A (2021) Hydroxychloride trace minerals have a positive effect on growth performance, carcass quality and impact ileal and cecal microbiota in broiler chickens. J Anim Sci Biotechnol 12:38. 10.1186/s40104-021-00553-7 33685507 10.1186/s40104-021-00553-7 PMC7941882

[31] Ghasemi HA, Hajkhodadadi I, Hafizi M, Fakharzadeh S, Abbasi M, Kalanaky S, Nazaran MH (2022) Effect of advanced chelate compounds-based mineral supplement in laying hen diet on the performance, egg quality, yolk mineral content, fatty acid composition, and oxidative status. Food Chem 366:130636. 10.1016/j.foodchem.2021.130636 34314929 10.1016/j.foodchem.2021.130636

[32] Saleh AA, Eltantawy MS, Gawish EM, Younis HH, Amber KA, Abd El-Moneim AEME, Ebeid TA (2020) Impact of dietary organic mineral supplementation on reproductive performance, egg quality characteristics, lipid oxidation, ovarian follicular development, and immune response in laying hens under high ambient temperature. Biol Trace Elem Res 195:506–514 (2020). 10.1007/s12011-019-01861-w 10.1007/s12011-019-01861-w 31418151

[33] Ivanišinová O, Grešáková Ľ, Ryzner M, Oceľová V, Čobanová K (2016) Effects of feed supplementation with various zinc sources on mineral concentration and selected antioxidant indices in tissues and plasma of broiler chickens. Acta Vet Brno 85(3):285–291. 10.2754/avb201685030285

[34] Pérez V, Shanmugasundaram R, Sifri M, Parr TM, Selvaraj RK (2017) Effects of hydroxychloride and sulfate form of zinc and manganese supplementation on superoxide dismutase activity and immune responses post lipopolysaccharide challenge in poultry fed marginally lower doses of zinc and manganese. Poult Sci 96:4200–4207. 10.3382/ps/pex244 29053870 10.3382/ps/pex244

[35] Ülger İ, Mahmood SS (2023) Comparison of plant extract-derived nano-zinc particles with different zinc sources and effects of different zn sources on egg yield and quality traits. Anim Sci J 94:e13903. 10.1111/asj.13903 38115216 10.1111/asj.13903

